# Alterations of nitric oxide homeostasis as trigger of intestinal barrier dysfunction in non‐alcoholic fatty liver disease

**DOI:** 10.1111/jcmm.17175

**Published:** 2022-01-14

**Authors:** Anja Baumann, Dragana Rajcic, Annette Brandt, Victor Sánchez, Finn Jung, Raphaela Staltner, Anika Nier, Michael Trauner, Katharina Staufer, Ina Bergheim

**Affiliations:** ^1^ Department of Nutritional Sciences Molecular Nutritional Science University of Vienna Vienna Austria; ^2^ Division of Gastroenterology & Hepatology Department of Internal Medicine III Medical University of Vienna Vienna Austria; ^3^ Department of Surgery Division of Transplantation Medical University of Vienna Vienna Austria

**Keywords:** arginase activity, endotoxin, intestinal barrier function, L‐arginine, non‐alcoholic steatohepatitis

## Abstract

Changes in intestinal nitric oxide metabolism are discussed to contribute for the development of intestinal barrier dysfunction in non‐alcoholic fatty liver disease (NAFLD). To induce steatosis, female C57BL/6J mice were pair‐fed with a liquid control diet (C) or a fat‐, fructose‐ and cholesterol‐rich diet (FFC) for 8 weeks. Mice received the diets ± 2.49 g L‐arginine/kg bw/day for additional 5 weeks. Furthermore, mice fed C or FFC ± L‐arginine/kg bw/day for 8 weeks were concomitantly treated with the arginase inhibitor N^ω^‐hydroxy‐nor‐L‐arginine (nor‐NOHA, 0.01 g/kg bw). Liver damage, intestinal barrier function, nitric oxide levels and arginase activity in small intestine were assessed. Also, arginase activity was measured in serum from 13 patients with steatosis (NAFL) and 14 controls. The development of steatosis with beginning inflammation was associated with impaired intestinal barrier function, increased nitric oxide levels and a loss of arginase activity in small intestine in mice. L‐arginine supplementation abolished the latter along with an improvement of intestinal barrier dysfunction; nor‐NOHA attenuated these effects. In patients with NAFL, arginase activity in serum was significantly lower than in healthy controls. Our data suggest that increased formation of nitric oxide and a loss of intestinal arginase activity is critical in NAFLD‐associated intestinal barrier dysfunction.

## 1 INTRODUCTION

Non‐alcoholic fatty liver disease (NAFLD) has become the most commonly diagnosed liver disease worldwide affecting approximately a quarter of the general population.^1^ Recently, it has been reported that liver cirrhosis due to non‐alcoholic steatohepatitis (NASH), as a progressive variant of NAFLD, has become the leading cause on the liver transplantation list in US adults.^2^ In spite of these concerning numbers, the underlying molecular mechanisms have not yet been clarified and universally acknowledged prevention and treatment options are restricted to diet and exercise. In more recent years, results of animal and human studies indicate that besides diet and physical inactivity, dysfunction of intestinal barrier and subsequently, an increased translocation of bacterial endotoxin may also be among the key factors in the onset and the progression of NAFLD.^3^, ^4^, ^5^ Indeed, it has been shown that plasma bacterial endotoxin levels in portal vein are elevated and expression of toll‐like receptor 4 (*Tlr4*), as well as dependent signalling cascades in liver, are induced in patients with NAFLD.^6^, ^7^, ^8^ Additionally, results of animal models of NAFLD targeting endotoxin‐dependent signalling cascades, and even more so, intestinal barrier function,^3^, ^5^, ^9^ suggest that intestinal barrier dysfunction is critical in the development of the disease.

Alterations of nitric oxide bioavailability in intestinal epithelial cells have been discussed to be critical in maintaining the intestinal epithelial barrier structure.^10^ Indeed, while moderate amounts of nitric oxide are continuously produced by the neuronal and endothelial nitric oxide synthase (NOS1, NOS3) in the intestinal mucosa, being critical in maintaining intestinal homeostasis and integrity, an induction of inducible nitric oxide synthase (iNOS, NOS2) has been proposed to be associated with intestinal barrier dysfunction in various settings including NAFLD in humans.^11^, ^12^ In support of these findings, it has been shown that supplementation of the amino acids L‐citrulline and L‐arginine, shown to restore intracellularly the nitric oxide formation, suppress iNOS activity in the intestine in the presence of endotoxaemia in rodents.^13^ However, the underlying molecular mechanism of the diet‐induced disruption of intestinal barrier function in mice with diet‐induced NAFLD and the role of nitric oxide, herein, have not yet been fully clarified. Based on this background, the aim of the study was to assess whether changes in intestinal nitric oxide and L‐arginine metabolism are critical in the development of diet‐induced intestinal barrier dysfunction in mice with NAFLD and if targeting these changes through an oral supplementation of L‐arginine or the arginase inhibitor N^ω^‐hydroxy‐nor‐L‐arginine (nor‐NOHA) alters the progression of steatosis to later disease stages, for example NASH.

## 2 MATERIALS AND METHODS

### 2.1 Animals and intervention trials

Six‐eight weeks old female C57BL/6J mice (Janvier SAS, Le Genest‐Saint‐Isle, France) were housed in controlled conditions in a specific pathogen‐free barrier facility accredited by the Association for Assessment and Accreditation of Laboratory Animal Care. All procedures and treatments were approved by the local institutional animal care and use committee (Landesamt für Verbraucherschutz, Thuringia, Germany and Federal Ministry Republic of Austria Education, Science and Research, Vienna, Austria). Mice had free access to water at all times and were housed in groups. It has been shown that female C57BL/6J mice were more susceptible to fructose‐induced NAFLD^14^ and results of a meta‐analysis suggest that variability of traits and parameters is similar between male and female mice.^15^ For the ex‐vivo‐everted gut sac experiments, naïve female C57BL/6J mice were sacrificed via cervical dislocation. For feeding experiments, animals were randomly assigned to the following feeding groups (the study designs are summarized in Figure S1). The calculation of sample size was based on previous findings.^3^, ^16^ Intervention trial 1: Mice were fed a liquid control diet (C; 15.7 MJ/kg diet: 69E% carbohydrates, 12E% fat, 19E% protein; Ssniff) or a liquid fat‐, fructose‐ and cholesterol‐rich diet (FFC; 17.8 MJ/kg diet: 60E% carbohydrates, 25E% fat, 15E% protein with 50% wt/wt fructose and 0.16% wt/wt cholesterol; Ssniff) as detailed previously.^3^ After an adaption phase to the liquid diet, mice were pair‐fed C or FFC for 8 weeks. In week 8, tissue and blood were collected from some mice fed C or FFC as detailed below. Remaining animals were randomized to the following groups (*n* = 6–8/group): mice fed liquid C ± 2.49 g L‐arginine/kg bw/day (C, C + Arg) and mice fed FFC ± 2.49 g L‐arginine/kg bw/day (FFC, FFC + Arg) for additional 5 weeks. Intervention trial 2: Furthermore, 6–8 weeks old female C57BL/6J mice (*n* = 6/group) were fed drinking water enriched with 30% (w/v) fructose (F) in addition to standard chow for 16 weeks or plain water. For details regarding feeding and liver damage and markers of intestinal permeability see also Sellmann et al.^17^ Intervention trial 3: After the adaption to the liquid diets, mice were pair‐fed C or FFC supplemented with a mixture of non‐resorbable antibiotics (AB) for 8 weeks resulting in the following experimental groups: C, FFC or FFC + AB. AB mixtures consisted of polymyxin (92 mg/kg bw/day) and neomycin (216 mg/kg bw/day) as detailed before.^18^ Intervention trial 4: Once adaption to the liquid diet, animals were pair‐fed a liquid C or FFC ± 2.49 g L‐arginine/kg bw/day and were treated i.p. with the arginase inhibitor nor‐NOHA (0.01 g/kg bw; Bachem AG) or vehicle 3 times per week for 8 weeks resulting in the following experimental groups: C, FFC, FFC + NOHA, FFC + Arg and FFC + Arg + NOHA.

At the end of the trials, mice were anaesthetized with 100 mg ketamine/kg bw and 16 mg xylazine/kg bw. Blood from portal vein was collected just prior to cervical dislocation. Blood, livers and intestinal tissue were collected to determine markers of liver damage and intestinal barrier function. Therefore, tissue was fixed in neutral‐buffered formalin or snap‐frozen for further analyses.

### 2.2 Everted sac model of mice ex vivo

Small intestines (*n* = 7/treatment) from naïve female C57BL/6J mice were collected and everted with a rod as previously described,^19^, ^20^ cut into equal sections, ligated at both ends and filled with 1X Krebs‐Henseleit‐bicarbonate buffer supplemented with 0.2% bovine serum albumin (KRH buffer). Everted gut sacs were preincubated in gassed (95% O_2_/5% CO_2_) KRH buffer (Ctr) ± 0.04 mM L‐arginine at 37°C for 10 min and then further incubated with 5 mM fructose (F) ± 0.04 mM L‐arginine (Arg + F), respectively, at 37°C for 1 h. To determine tissue permeability, everted small intestinal tissue sacs were incubated in 0.1% D‐xylose (Sigma‐Aldrich Chemie) for 5 min in above‐mentioned incubation solutions, subsequently. After incubation, liquids inside the everted tissue sacs were collected, and intestinal tissue was snap‐frozen until further analysis.

### 2.3 Human study

Serum was collected from 13 patients with steatosis (NAFL patients) as diagnosed by ultrasound and/or liver biopsies and 14 age‐matched healthy controls. All procedures were approved by the ethics committee of the Medical University of Vienna and informed consent was obtained from all subjects before the study (747/2011). Characteristics of the NAFL patients and controls and liver histology of NAFL patients are summarized in Table S1.

### 2.4 Histological evaluation of liver and hepatic triglyceride accumulation

Paraffin‐embedded liver sections (4 µm) of mice were stained with haematoxylin and eosin (Sigma‐Aldrich Chemie) and evaluated using NAFLD activity score (NAS) adapted from Kleiner et al.^21^ Neutrophilic granulocytes were stained using a commercially available Naphthol AS‐D Chloroacetate Specific Esterase Kit (Sigma‐Aldrich Chemie) as described before.^14^ Concentration of hepatic triglycerides was measured in liver homogenates using a commercially available kit (Randox Laboratories).

### 2.5 Blood parameters of liver damage

Activities of alanine aminotransferase (ALT) and aspartate aminotransferase (AST) in mouse plasma were measured in a routine laboratory (Friedrich‐Alexander University).

### 2.6 Arginase activity and nitrite (NO_2_^−^) measurement

Arginase activity was measured in proximal intestinal tissue of mice and in serum of NAFL patients and healthy controls as detailed previously.^22^ To determine arginase activity in tissue samples, proximal small intestine was homogenized in 10 mM Tris‐HCl containing 0.4% (w/v) Triton X‐100 and protease inhibitor cocktail. Levels of NO_2_^−^ in proximal small intestine were detected using Griess assay (Promega).

### 2.7 D‐xylose assay

To determine tissue permeability, xylose concentration in collected liquids of everted gut sacs was measured using a commercially available kit (Megazyme, Bray).

### 2.8 Endotoxin, TLR4 ligand measurement, myeloperoxidase (MPO) assay and ELISA

Bacterial endotoxin levels in mouse portal plasma were determined using a limulus amebocyte lysate assay (Charles River) as reported in detail previously.^3^ TLR4 ligands in mouse portal plasma and human serum were measured as previously described^23^ using commercially available reporter gene assays with TLR4 transfected HEK293 cells following the instructions of the manufacturer (InvivoGen). For measuring MPO, liver tissue was homogenized in phosphate buffer and determined as detailed previously.^3^ Plasminogen activator inhibitor 1 (PAI‐1) concentrations in livers were measured with a commercially available kit (LOXO).

### 2.9 Immunohistochemical staining

Paraffin‐embedded liver sections (4 µm) were stained for iNOS (Thermo Fisher Scientific) and F4/80‐positive cells (F4/80 antibody: Abcam) as described previously.^19^ Furthermore, paraffin‐embedded intestinal sections (4 µm) were stained with anti‐zonula occludens 1 (ZO‐1; Invitrogen) as described previously.^5^ Staining was evaluated using an analysis system (Leica Applications Suite) incorporated in a microscope (Leica).

### 2.10 RNA isolation, real‐time RT‐PCR

Total RNA from liver was extracted with Trizol (peqGOLD TriFast, Peqlab). cDNA was synthetized with a reverse transcription system (Promega) and real‐time PCR was performed using iTaq^TM^ Universal SYBR Green Supermix (Bio‐Rad Laboratories). Table S2 summarizes the primer sequences that were used.

### 2.11 Western Blot analysis

Intestinal tissue was homogenized in RIPA buffer containing protease inhibitor cocktail (Sigma‐Aldrich Chemie) and protein lysates were transferred to a polyvinylidene difluoride membrane (Bio‐Rad Laboratories) probed with primary antibodies against arginase‐1 (ARG‐1), arginase‐2 (ARG‐2), occludin, myosin light chain kinase (MYLK) or β‐actin (ARG‐1, ARG‐2 and β‐actin: Cell Signaling Technology; occludin and MYLK: Invitrogen) and secondary antibodies (Cell Signaling Technology). Intensities of bands were detected with Super Signal West Dura Kit (Thermo Fisher Scientific) and analysed using a ChemiDoc XRS System.

### 2.12 Statistics

Data are presented as means ± standard error of the means (SEM). Statistical analyses were performed with GraphPad Prism Version 7.0. Grubbs test was used to identify outliers. A Student *t* test was used to analyse differences between C‐ and FFC‐fed animals fed for 8 weeks, C and F‐fed animals fed for 16 weeks, or for analysis between NAFL patients and healthy controls. A one‐way ANOVA and two‐way ANOVA, respectively, were applied to determine statistical differences between three and more different treatment groups. For analysing parameters obtained from ex‐vivo‐everted gut sac experiments, one‐way ANOVA was used. A *p*‐value <0.05 was defined as significant.

## 3 RESULTS

### 3.1 Markers of liver damage, TLR4 signalling cascade and intestinal barrier dysfunction in FFC‐fed mice

**FIGURE 1:**
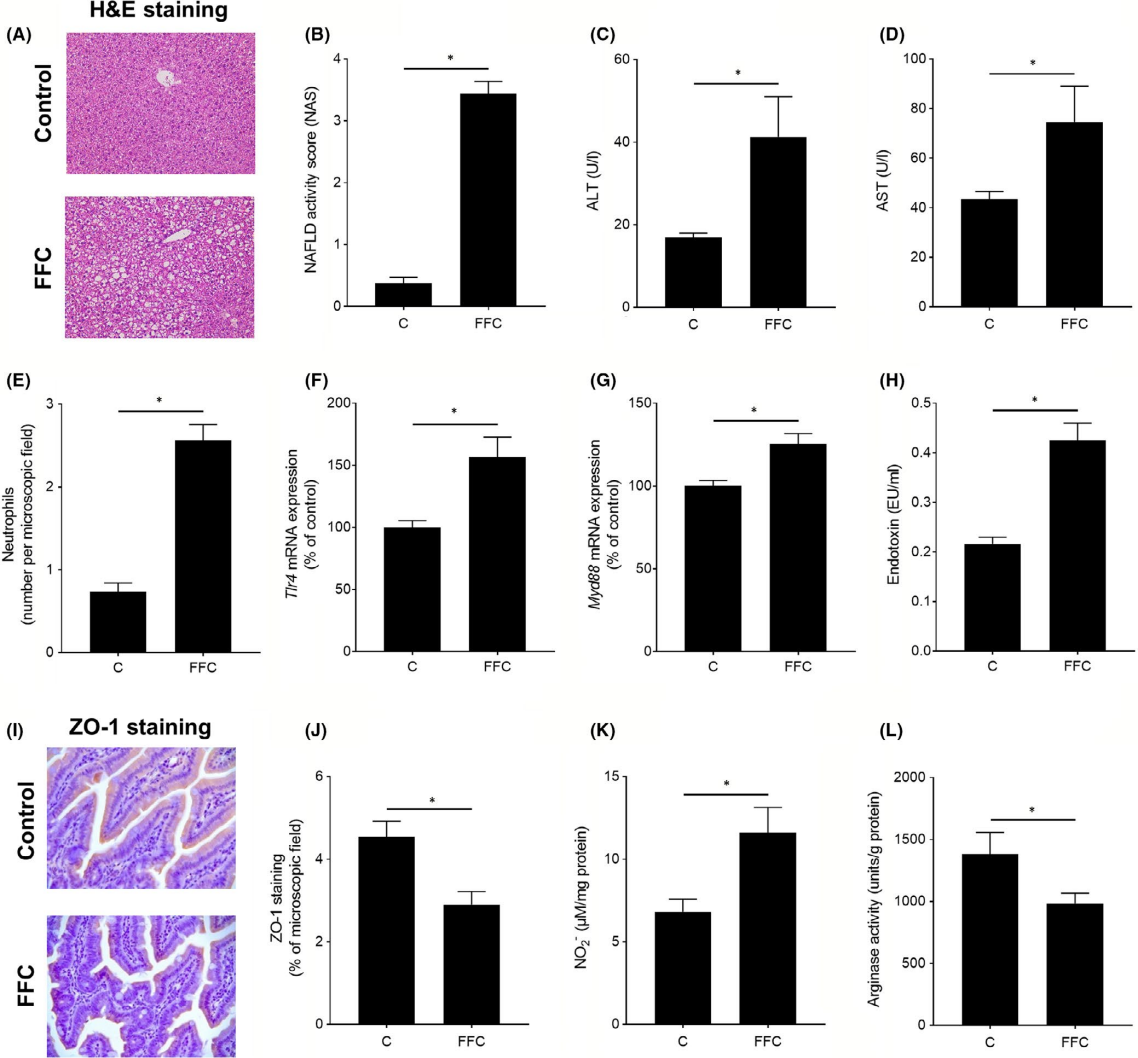
Indices of liver damage, markers of intestinal barrier function and NO_2_^−^ concentration in FFC‐fed C57BL/6J mice. (A) Representative pictures of haematoxylin and eosin (H&E) staining of livers (magnification 200X), (B) evaluation of NAFLD activity score (NAS), activities of (C) alanine aminotransferase (ALT) and (D) aspartate aminotransferase (AST) in plasma, (E) number of neutrophils in liver tissue, mRNA expression of hepatic (F) toll‐like receptor 4 (*Tlr4*) and (G) myeloid differentiation primary response 88 (*Myd88*), (H) plasma endotoxin levels, (I,J) representative photomicrographs (magnification 400X) and densitometric analysis of zonula occludens 1 (ZO‐1) staining and (K) nitrite (NO_2_^−^) concentration in proximal small intestinal tissue as well as (L) arginase activity in duodenal tissue in C‐ and FFC‐fed mice. Data are shown as means ± SEM, *n* = 4–8, *p < 0.05. C, control diet; FFC, fat‐, fructose‐ and cholesterol‐rich diet; NAFLD, non‐alcoholic fatty liver disease

After 8 weeks of feeding, FFC‐fed mice had developed obvious signs of steatosis and beginning hepatic inflammation with the higher number of infiltrating neutrophils, NAS and plasma activities of ALT and AST (*p* < 0.05, Figure 1A‐E). Expression of *Tlr4* and myeloid differentiation primary response 88 (*Myd88*) mRNA in liver tissue, as well as bacterial endotoxin levels in portal plasma, being all indicative of intestinal barrier dysfunction,^19^, ^22^ were also significantly higher in mice fed the FFC in comparison to C‐fed mice (Figure 1F‐H). In addition, ZO‐1 protein levels in small intestinal tissue were significantly lower in FFC‐fed mice compared to C‐fed mice (Figure 1I,J). The loss of ZO‐1 was associated with higher levels of NO_2_^−^ in small intestine in FFC‐fed mice (Figure 1K). Furthermore, arginase activity, which is suggested to be the opponent of iNOS in the balance of nitric oxide availability, and subsequently, pro‐ and anti‐inflammatory processes,^24^, ^25^ was significantly lower in small intestine of FFC‐fed mice than in controls (Figure 1L). Interestingly, protein levels of ARG‐2 found to be the predominant arginase isotype in small intestine,^26^ were similar between groups while ARG‐1 protein was not detectable in small intestinal tissue (Figure S2). In line with these findings, alterations of markers of intestinal barrier function, for example elevation of portal endotoxin levels and decreased tight junctions protein levels in mice suffering from fructose‐induced NAFLD, was also associated with significantly lower arginase activity compared to mice fed plain water (Figure S3, for further details regarding the experimental set‐up and markers of intestinal barrier function see Sellmann et al.^17^).

**FIGURE 2:**
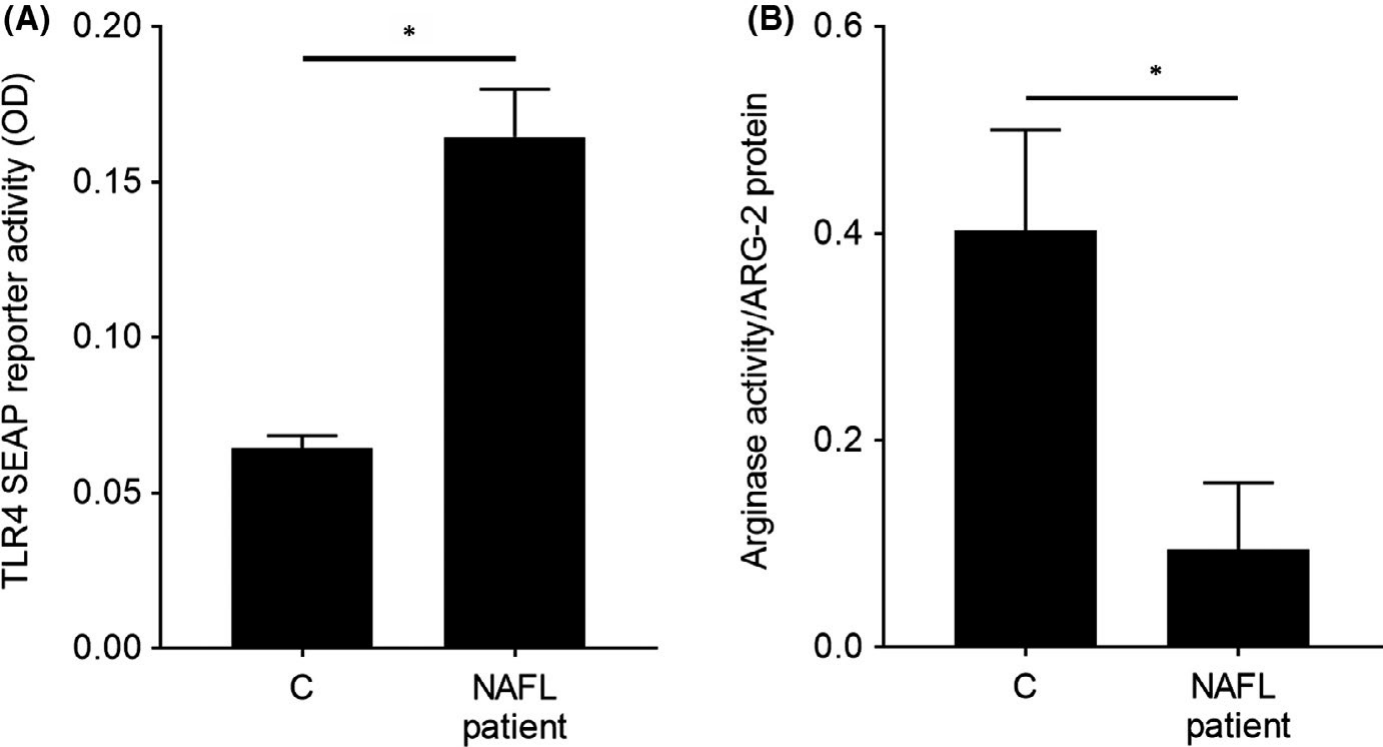
Marker of intestinal barrier function and arginase activity in NAFL patients. (A) TLR4 SEAP reporter activity in serum and (B) arginase activity related to arginase‐2 (ARG‐2) protein in serum of NAFL patients and healthy controls. Data are shown as means ± SEM, *n* = 13–14 except for (B): *n* = 5–10 as values were in part below the level of detection, *p < 0.05. C, healthy control; NAFL patients, patients with steatosis; SEAP, secreted embryonic alkaline phosphatase

To elucidate if alterations alike are also associated with the development of NAFLD in humans, markers of intestinal permeability and arginase activity were determined in serum of NAFL patients and controls. In line with the findings in mice, marker of intestinal permeability in serum, like TLR4 ligands were higher in NAFL patients than in healthy probands, while arginase activity was significantly lower (*p* < 0.05, Figure 2A,B).

### 3.2 Effect of antibiotics on intestinal permeability and arginase activity in small intestine in FFC‐fed mice

**FIGURE 3:**
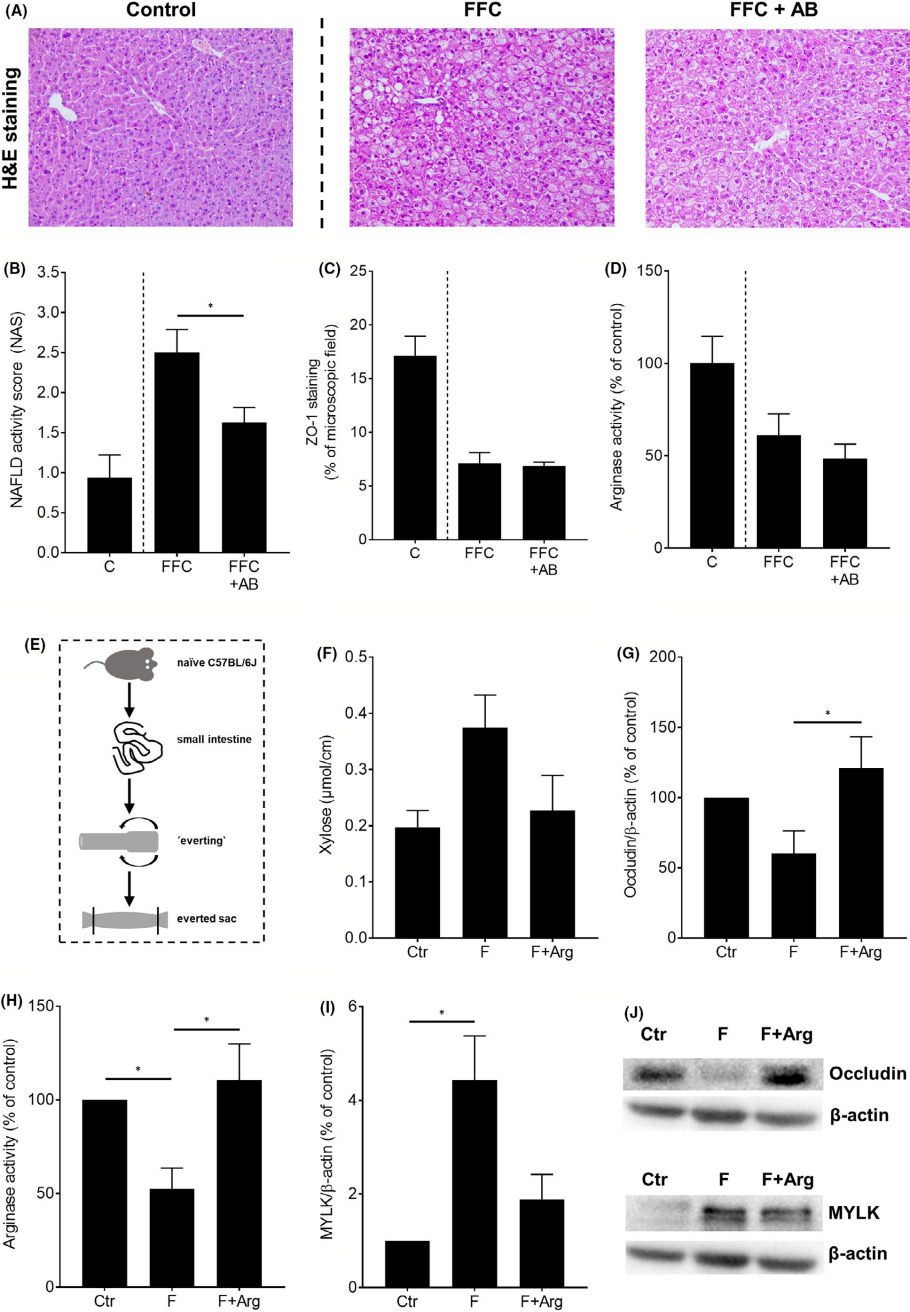
Effect of antibiotic treatment on liver status, tight junction proteins and arginase activity in small intestine of FFC‐fed C57BL/6J mice as well as effect of L‐arginine on markers of intestinal barrier function and arginase activity in everted gut sac model ex vivo. (A) Representative pictures of haematoxylin and eosin (H&E) staining of liver sections (magnification 200X), (B) NAFLD activity score (NAS), (C) densitometric analysis of zonula occludens 1 (ZO‐1) staining and (D) arginase activity in proximal small intestinal tissue of FFC‐fed mice. (E) Schematic drawing of everted sac experiment ex vivo, (F) xylose permeation, (G) protein concentration of occludin, (H) arginase activity, (I) protein concentration of myosin light chain kinase (MYLK) and (J) representative blots of occludin, MYLK and β‐actin in whole intestinal tissue specimen obtained from naïve mice of everted gut experiments treated with 5 mM fructose supplemented with 0.04 mM L‐arginine. Data are shown as means ± SEM, for mice experiments: *n* = 5–8, for everted sac experiments: *n* = 4–7, *p < 0.05. AB, antibiotics; Arg, L‐arginine; C, control diet; Ctr, everted gut sacs incubated in 1x Krebs‐Henseleit‐bicarbonate buffer; F, fructose; FFC, fat‐, fructose‐ and cholesterol‐rich diet; NAFLD, non‐alcoholic fatty liver disease

To determine whether the alterations found in small intestinal tissue of FFC‐fed mice were related to intestinal microbiota, mice pair‐fed the FFC were concomitantly treated with the non‐resorbable antibiotics polymyxin B and neomycin. C‐fed mice are shown for comparison. As expected, the development of NAFLD was significantly attenuated in mice fed the FFC + AB as determined by NAS (Figure 3A,B). Despite the similar caloric intake, absolute body‐ and liver weight and liver to body weight ratio, AST activities in plasma were significantly lower in FFC + AB‐fed mice compared to FFC‐fed mice (Table S3). In contrast, protein concentrations of the tight junction ZO‐1 in small intestinal tissue were similar between the two FFC‐fed groups and markedly lower than in C‐fed mice (Figure 3C; Figure S4). Furthermore, arginase activity in small intestinal tissue was also similar in both FFC‐fed groups. However, arginase activity was markedly lower than in controls (Figure 3D).

### 3.3 Effect of fructose on arginase activity and markers of intestinal permeability in everted gut sacs of mice

To assess if a loss of arginase activity plays an important role in the development of intestinal barrier dysfunction in NAFLD and if fructose found in diet is critical; herein, everted gut sacs of naïve mice were incubated with fructose. The ex‐vivo‐everted sac technique is summarized in Figure 3E. Permeation of xylose was by trend higher in small intestine of everted sacs incubated with fructose, while protein levels of occludin and arginase activity were lower in small intestine of these sacs. However, as levels of xylose and occludin protein varied considerably between tissue sacs, only the results of arginase activity reached the level of significance. Protein concentration of MYLK was significantly higher in fructose challenged intestinal tissue of everted sacs compared to everted gut sacs only incubated in KRH buffer. These changes were almost completely diminished when small intestinal tissue of everted gut sacs were additionally incubated with 0.04 mM L‐arginine, which was shown to be an allosteric regulator of arginase (Figure 3F‐J).

### 3.4 Effect of L‐arginine on markers of liver damage and intestinal barrier function in FFC‐fed mice

**FIGURE 4:**
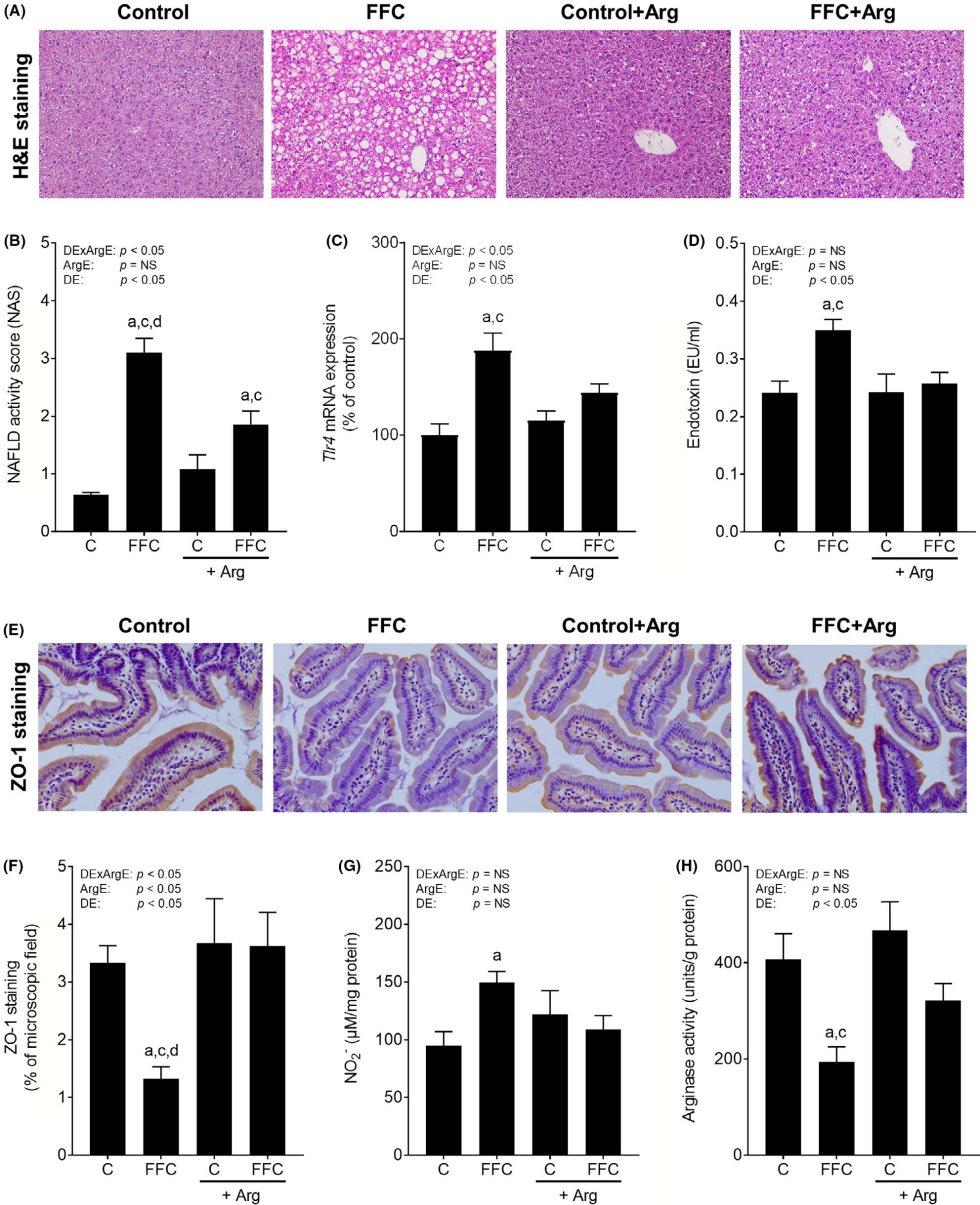
Effect of an oral supplementation of L‐arginine on liver damage as well as markers of intestinal permeability in C57BL/6J mice with FFC‐induced NAFLD. (A) Representative picture of haematoxylin and eosin (H&E) staining of liver tissue (magnification 200X), (B) evaluation of NAFLD activity score (NAS), (C) mRNA expression of hepatic toll‐like receptor 4 (*Tlr4*), (D) plasma endotoxin levels, (E,F) representative picture (magnification 400X) and densitometric analysis of zonula occludens 1 (ZO‐1) staining in small intestinal tissue. (G) Nitrite (NO_2_^−^) concentration and (H) arginase activity in small intestinal tissue. Data are shown as means ± SEM, *n* = 4–8, except for (G): *n* = 3 for mice fed C + Arg diet, ^a^ *p* < 0.05 compared with mice fed a C diet, ^c^ *p* < 0.05 compared with mice fed a C+Arg diet, ^d^ *p* < 0.05 compared with mice fed an FFC + Arg diet. ArgE, L‐arginine effect; C, control diet; DE, diet effect; DExArgE, interaction between diet and L‐arginine effect; FFC, fat‐, fructose‐ and cholesterol‐rich diet; NAFLD, non‐alcoholic fatty liver disease; NS, not significant

To determine whether targeting intestinal arginase activity has a beneficial effect on intestinal barrier dysfunction in vivo, mice with diet‐induced NAFLD were either pair‐fed the FFC or an FFC supplemented with L‐arginine for 5 weeks. In accordance with previous findings,^16^ supplementation of L‐arginine significantly attenuated the progression of NAFLD in mice with pre‐existing NAFLD despite the continuous feeding of the FFC. Indeed, while still being in part higher than in controls, NAS, infiltration of neutrophils and F4/80‐positive cells as well as iNOS protein levels in liver tissue were significantly lower in livers of FFC + Arg‐fed mice when compared to FFC‐fed animals (Figure 4A,B; Table 1). However, liver weight and liver to body weight ratio were still significantly higher in both FFC‐fed groups compared to controls (Table 1). Interleukin 6 (*Il6*) mRNA expression in livers of FFC‐fed mice were significantly or by trend higher compared to both control groups (FFC vs. C: *p* = 0.05 and FFC vs. C + Arg: *p* < 0.05), while differences alike were not found when comparing control groups with FFC + Arg‐fed animals. Expression of tumour necrosis factor‐alpha (*Tnfα*) mRNA and PAI‐1 protein levels in livers of both FFC‐, and FFC + Arg‐fed mice were significantly or by trend higher than in both control groups. Furthermore, as data varied considerably, ALT and AST activity levels were similar between groups (Table 1).

**TABLE 1:** Effect of an oral supplementation of L‐arginine on caloric intake, parameters of liver damage and inflammation in C57BL/6J mice with FFC‐induced NAFLD

	Diet groups (13 weeks)				*p* (two‐way ANOVA)		
	C	FFC	C + Arg	FFC + Arg	DEx ArgE	ArgE	DE
Caloric intake (kcal/g bw)	0.46 ± 0.0	0.46 ± 0.0	0.48 ± 0.0	0.48 ± 0.0	NS	<0.05	NS
Body weight (g)	23.4 ± 1.0	22.9 ± 0.2^c^	25.1 ± 0.5	23.0 ± 0.3	NS	NS	<0.05
Liver weight (g)	1.1 ± 0.1	1.5 ± 0.0^a^, ^c^	1.2 ± 0.0	1.5 ± 0.1^a^, ^c^	NS	NS	<0.05
Liver:body weight ratio (%)	4.7 ± 0.1	6.8 ± 0.1^a^, ^c^	5.0 ± 0.1	6.6 ± 0.1^a^, ^c^	<0.05	NS	<0.05
Triglycerides (μg/mg protein)	38.5 ± 3.7	110.8 ± 4.0^a^, ^c^	32.5 ± 6.6	103.4 ± 8.1^a^, ^c^	NS	NS	<0.05
ALT (U/L)	23.1 ± 4.6	36.6 ± 2.9	29.6 ± 3.6	27.0 ± 3.3	<0.05	NS	NS
AST (U/L)	51.9 ± 6.0	77.6 ± 7.8	63.3 ± 6.6	58.1 ± 6.2	<0.05	NS	NS
Neutrophils^e^	1.9 ± 0.1	3.4 ± 0.2^a^, ^c^, ^d^	1.8 ± 0.1	2.6 ± 0.3^a^, ^c^	NS	<0.05	<0.05
F4/80‐positive cells^e^	13.4 ± 0.7	19.1 ± 1.3^a^, ^c^, ^d^	12.7 ± 0.4	14.3 ± 0.3	<0.05	<0.05	<0.05
iNOS protein concentration^g^	5.4 ± 1.1	17.8 ± 1.7^a^, ^c^, ^d^	5.5 ± 0.8	11.4 ± 1.4^a^, ^c^	<0.05	<0.05	<0.05
*Il6* mRNA expression^f^	100.0 ± 13.9	194.2 ± 36.9^c^	87.3 ± 20.5	112.4 ± 24.0	NS	NS	<0.05
PAI−1^f^	100.0 ± 7.9	149.0 ± 16.5^a^, ^c^	84.7 ± 8.4	140.8 ± 8.3^c^	NS	NS	<0.05
*Tnfa* mRNA expression^f^	100.0 ± 12.5	281.9 ± 44.4^a^, ^c^	86.9 ± 18.4	209.2 ± 24.3^a^	NS	<0.05	<0.05

Data are shown as means ± SEM, *n* = 5–8.

Abbreviations: ALT, alanine aminotransferase; Arg, L‐arginine; ArgE, L‐arginine effect; AST, aspartate aminotransferase; C, control diet; DE, diet effect; DExArgE, interaction between diet and L‐arginine effect; FFC, fat‐, fructose‐ and cholesterol‐rich diet; Il, interleukin; iNOS, inducible nitric oxide synthase; NS, not significant; PAI‐1, plasminogen activator inhibitor 1; Tnfα, tumour necrosis factor‐alpha.

^a^ *p* < 0.05 compared with mice fed a C diet.

^c^ *p* < 0.05 compared with mice fed a C + Arg diet.

^d^ *p* < 0.05 compared with mice fed an FFC + Arg diet.

^e^ Number per microscopic field.

^f^ Per cent of control.

^g^ Per cent of microscopic field.

The progression of NAFLD was associated with a significant higher *Tlr4* mRNA expression in liver tissue and higher bacterial endotoxin levels in portal plasma of FFC‐fed animals when compared to both control groups (Figure 4C,D). Differences alike were not found when comparing *Tlr4* expression and portal endotoxin levels between FFC + Arg and control groups. ZO‐1 protein concentrations in small intestinal tissue were significantly lower in FFC‐fed mice compared to C‐fed and FFC + Arg‐fed mice. Again, differences alike were not found between FFC + Arg‐fed mice and controls (Figure 4E,F). Beneficial effects of the L‐arginine supplementation in FFC‐fed mice on changes in markers of intestinal barrier function were associated with an alleviation of the significant increases of NO_2_^−^ concentrations and the significant loss of arginase activity in small intestinal tissue found in FFC‐fed mice (Figure 4G,H). Again, protein levels of ARG‐2 were not different between groups (Figure S5).

### 3.5 Effect of inhibiting arginase activity on the development of liver damage and markers of intestinal barrier function in FFC‐fed mice

**FIGURE 5:**
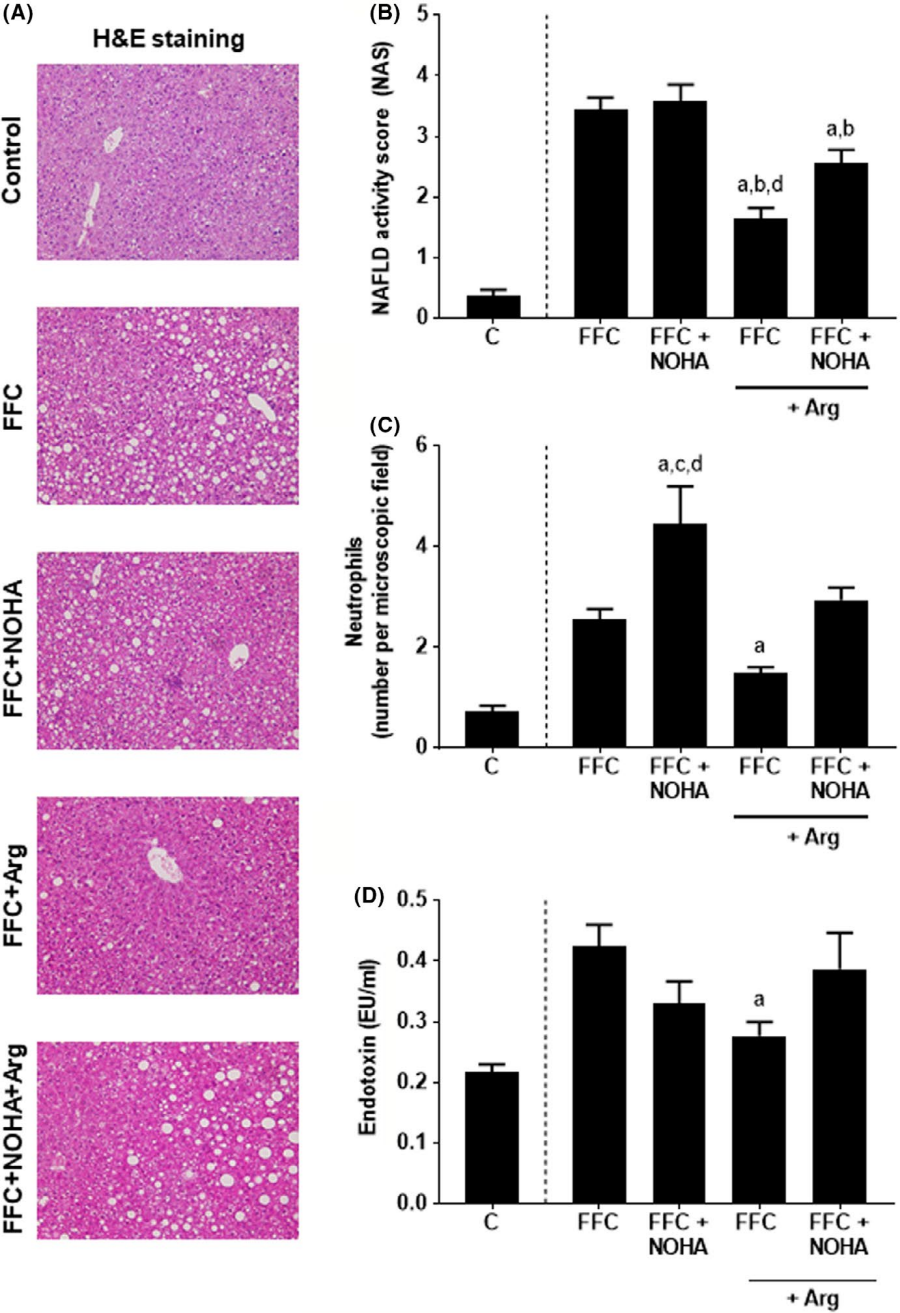
Effect of an oral supplementation of L‐arginine and nor‐NOHA treatment on liver damage and portal endotoxin levels in FFC‐fed C57BL/6J mice. (A) Representative pictures of haematoxylin and eosin (H&E) staining of livers (magnification 200X), (B) evaluation of NAFLD activity score (NAS), (C) number of neutrophils in liver tissue and (D) plasma endotoxin levels. Data are shown as means ± SEM, *n* = 6–8, ^a^ *p* < 0.05 compared with mice fed an FFC diet, ^b^ *p* < 0.05 compared with mice fed an FFC diet and treated with NOHA, ^c^ *p* < 0.05 compared with mice fed an FFC + Arg diet, ^d^ *p* < 0.05 compared with mice fed an FFC + Arg diet and treated with NOHA. Arg, L‐arginine; C, control diet; FFC, fat‐, fructose‐ and cholesterol‐rich diet; NOHA, N^ω^‐hydroxy‐nor‐L‐arginine

To further delineate if a loss of arginase activity is an important factor in the development of NAFLD, mice were additionally treated with the arginase inhibitor nor‐NOHA while being fed the C‐ and FFC diet supplemented −/+ L‐arginine for 8 weeks. No differences were found between the different control groups; therefore, results of C‐fed mice are shown as representative of all control groups. Despite similar caloric intake, markers of liver damage, for example NAS, liver weight and liver to body weight ratio were significantly higher in FFC‐ and FFC + NOHA‐fed mice when compared to FFC + Arg‐ and FFC + Arg + NOHA‐fed mice (Figure 5A,B; Table 2). Similar to the NAS, number of neutrophils was higher in all FFC‐fed groups than in controls. Number of neutrophils was also significantly higher in livers of FFC + NOHA‐fed mice than in all other groups, whereas number of neutrophils were similar between FFC‐ and FFC + NOHA + Arg‐fed mice. In FFC + Arg‐fed mice, number of neutrophils was significantly lower than in FFC‐fed mice (Figure 5C). The activity of MPO in liver was also higher in FFC‐fed groups than in C‐fed animals but did not differ between groups as data varied considerably within some groups (Table 2). In contrast, mRNA expression of *F4*/*80* in liver was similar between C‐ and FFC‐fed groups, while mRNA expression of *Tnfα* was higher in FFC‐fed groups regardless of additional treatments compared to controls; however, as data varied considerably in some groups, no differences were found between FFC groups (Table 2). Bacterial endotoxin levels in portal vein were elevated to a similar extent in FFC‐ and FFC + NOHA‐treated animals, whereas in FFC + Arg‐fed animals, bacterial endotoxin levels in portal plasma were significantly lower in comparison with FFC‐fed mice (Figure 5D). The treatment with nor‐NOHA attenuated these effects of L‐arginine supplementation in FFC‐fed mice.

**TABLE 2:** Effect of an oral supplementation of L‐arginine and nor‐NOHA treatment on caloric intake and parameters of liver damage and inflammation in C57BL/6J mice fed FFC

	Diet groups				
	C	FFC	FFC + NOHA	FFC + Arg	FFC + Arg + NOHA
Caloric intake (kcal/mouse/day)	8.8 ± 0.1	8.8 ± 0.1	8.8 ± 0.1	9.2 ± 0.1	9.2 ± 0.1
Body weight (g)	21.8 ± 0.4	21.6 ± 0.3	21.8 ± 0.2	20.8 ± 0.2	21.6 ± 0.3
Liver weight (g)	1.1 ± 0.0	1.5 ± 0.0	1.5 ± 0.0	1.2 ± 0.1^a^, ^b^	1.3 ± 0.1^a^, ^b^
Liver:body weight ratio (%)	4.9 ± 0.1	6.9 ± 0.1	6.9 ± 0.2	6.0 ± 0.2^a^, ^b^	5.9 ± 0.18^a^, ^b^
ALT (U/L)	16.9 ± 1.1	32.1 ± 4.0	36 ± 4.4	30.3 ± 3.5	40.9 ± 6.2
AST (U/L)	43.5 ± 3.1	74.6 ± 14.4	73.7 ± 3.6	71.8 ± 9.3	87.8 ± 15.2
MPO activity (% of control)	100.0 ± 5.5	277.5 ± 37.4	291.7 ± 35.0	198.1 ± 27.2	190.8 ± 16.2
*F4*/*80* mRNA expression^c^	100.0 ± 12.0	164.0 ± 7.6	109.8 ± 18.7	102.7 ± 15.3	108.7 ± 12.2
*Tnfa* mRNA expression^c^	100.0 ± 11.9	356.7 ± 100.6	210.1 ± 26.9	163.6 ± 22.1	156.7 ± 43.4

Data are shown as means ± SEM, *n* = 5–8.

Abbreviations: ALT, alanine aminotransferase; Arg, L‐arginine; AST, aspartate aminotransferase; C, control diet; FFC, fat‐, fructose‐ and cholesterol‐rich diet; MPO, myeloperoxidase; nor‐NOHA, N^ω^‐hydroxy‐nor‐L‐arginine, Tnfα, tumour necrosis factor‐alpha.

^a^ *p* < 0.05 compared with mice fed an FFC diet.

^b^ *p* < 0.05 compared with mice fed an FFC diet and treated with nor‐NOHA.

^c^ Per cent of control.

## 4 DISCUSSION

By now, results of many studies indicate that impairments of intestinal barrier function and elevated bacterial toxin levels in blood are critical contributors in the onset and progression of NAFLD (for overview see^27^). In support, it has been shown that in patients suffering from celiac disease, a disease associated with increased intestinal permeability, serum markers of NAFLD and ultrasound features of NAFLD are elevated.^28^ While studies suggest that diet, alterations of intestinal microbiota composition and physical activity as well as insulin resistance and overweight may be critical in the development of these changes, molecular mechanisms underlying intestinal barrier dysfunction in patients with NAFLD are still not fully clarified. Here, we showed that the development of NAFLD with early signs of inflammation in mice fed a high‐fructose, high‐fat and cholesterol‐rich diet even in the absence of overnutrition is associated with a loss of tight junction proteins and elevated levels of bacterial endotoxin in portal blood as well as an induction of the TLR4 signalling cascade in the liver. We further showed that the latter alterations were associated with higher NO_2_^−^ levels in small intestinal tissue and a loss of arginase activity of mice fed an FFC diet. Results of our own group and others have suggested before that a high intake of fructose both in humans and rodents is associated with increased levels of bacterial endotoxin and a loss of tight junction proteins in small intestine.^29^, ^30^, ^31^ The latter has been discussed to be dependent upon the changes in microbiota composition found to be associated with the intake of fructose‐rich diets.^5^, ^29^, ^32^ However, results of the present study suggest that the loss of tight junctions and changes of intestinal barrier function are independent of the presence of intestinal microbiota. Indeed, alterations alike were also found in the small intestine of FFC‐fed mice concomitantly treated with the non‐resorbable antibiotics polymyxin B and neomycin leading to a reduction of faecal microbiota of ~90% (data are not shown). This is in line with the findings of others showing that fructose can alter intestinal permeability even in the absence of microbiota and/or dysbiosis and at doses that can be considered as physiological relevant.^33^, ^34^ In summary, results of the present study suggest that fructose can alter the intestinal barrier function through microbiota‐independent mechanisms.

In support of the above, results of our ex vivo experiments suggest that fructose can induce intestinal barrier dysfunction in small intestinal tissue through direct effects. While it remains unclear through which exact metabolic signalling pathways fructose alters occludin and MYLK, both shown to be critical in maintaining tissue integrity and intestinal barrier function (for overview see^35^ and^36^), our data suggest that a loss of arginase activity plays an important role, herein. Indeed, concomitant treatment of everted gut sacs with L‐arginine abolished the effects of fructose on intestinal barrier function. Also, in patients with early stages of the disease, for example NAFL as diagnosed by liver histology, but signs of intestinal barrier dysfunction, arginase activity in serum was lower than in controls. Furthermore, in FFC‐fed mice, the protective effects of L‐arginine on the liver were also related to lower bacterial endotoxin levels in portal plasma and a lessened loss of tight junctions as well as a `normalization´ of NO_2_^−^ levels and arginase activity in small intestinal tissue. In addition, in FFC‐fed mice treated with nor‐NOHA, the beneficial effects of L‐arginine on liver tissue and bacterial endotoxin levels were almost completely attenuated. A role of arginase in intestinal barrier function, and even more so, in inflammation has been proposed by others before.^25^, ^37^, ^38^ For instance, it was shown that in humans and rodents with inflammatory bowel disease arginase‐1 expression is upregulated and may even correlate with the degree of inflammation.^25^, ^39^ Recently, it was shown that a genetic deletion of arginase‐1 in macrophages and endothelial cells is associated with a protection against intestinal inflammation^25^ and that this is also related to the arginine content in the diet. In the present study, ARG‐1 protein levels in small intestinal tissue were below the level of detection and there were no signs of inflammation prevalent in small intestines of FFC‐fed mice. Rather, results of the present study suggest that ARG‐2 is the isoform predominantly expressed in `normal´ small intestinal mucosa. However, as neither L‐arginine nor nor‐NOHA are specific activators or inhibitors of ARG‐2, respectively, it cannot be excluded, that ARG‐1 activity was also influenced by the interventions. Future studies employing more specific chemicals or knockout mice are needed to address the role of the different arginase isoforms.

An imbalance of iNOS and arginase has repeatedly been discussed to be related with impairments of intestinal integrity.^40^ Indeed, in‐vitro data from Talavera et al. suggest that in immunostimulated intestinal epithelial cells expression of both, NOS and arginase, was increased. In the same study, it was shown that an induction of arginase activity in enterocytes alleviates iNOS‐mediated nitric oxide production whereas the inhibition of arginase with nor‐NOHA resulted in enhanced nitric oxide production and decreases in viable cells.^40^ These data further suggest that an inhibition of iNOS and increase in arginase activity may be therapeutic strategies to prevent a loss of intestinal barrier function. In line with these findings, in the present study, the loss of arginase activity was associated with an elevation of NO_2_^−^ levels in small intestinal tissue, which was attenuated when arginase activity was induced. Taken together, the results of our study and those of others^40^ suggest that the loss of arginase activity, resulting in increased nitric oxide levels and through so far not fully understood mechanism in changes in MYLK and concentrations of tight junction proteins, may be key factors in the development of intestinal barrier dysfunction in the setting of NAFLD. Results of our study also suggest that dietary fructose intake may play a critical role herein and that an oral supplementation of L‐arginine can attenuate these alterations. Whether similar changes are also prevalent in humans with NAFLD and if they are related to fructose intake as well as molecular mechanisms involved need to be examined in the future research.

## 5 CONCLUSION

Our data suggest that the loss of intestinal barrier function in settings of diet‐induced NAFLD is associated with an increased formation of nitric oxide and a loss of arginase activity in small intestinal tissue. Our data further indicate, that herein, dietary fructose plays an important role and that the supplementation of L‐arginine can attenuate these alterations through `normalizing´ intestinal arginase activity. However, the underlying molecular mechanisms of the fructose‐dependent regulation of arginase activity, and subsequently, the attenuation or recovery of intestinal barrier function remain to be assessed in future research. Furthermore, while results of our study suggest that in humans with NAFLD, arginase activity in serum is also diminished while protein levels of arginase‐2 are unchanged, it remains to be determined if the formation of nitric oxide and arginase activity are also altered in small intestinal tissue of NAFLD patients and if this is related to (1) intestinal barrier dysfunction in these patients and (2) their consumption of fructose.

## CONFLICT OF INTERESTS

The authors declare that they have no conflict of interest.

## AUTHOR CONTRIBUTIONS

**Anja Baumann:** Data curation (equal); Formal analysis (equal); Investigation (equal); Visualization (equal); Writing – original draft (equal); Writing – review & editing (equal). **Dragana Rajcic:** Data curation (supporting); Formal analysis (supporting); Writing – review & editing (equal). **Annette Brandt:** Data curation (supporting); Formal analysis (supporting); Writing – review & editing (equal). **Victor Sánchez:** Data curation (supporting); Formal analysis (supporting); Writing – review & editing (equal). **Finn Jung:** Data curation (supporting); Formal analysis (supporting); Writing – review & editing (equal). **Raphaela Staltner:** Data curation (supporting); Formal analysis (supporting); Writing – review & editing (equal). **Anika Nier:** Data curation (supporting); Formal analysis (supporting); Writing – review & editing (equal). **Michael Trauner:** Data curation (supporting); Formal analysis (supporting); Writing – review & editing (equal). **Katharina Staufer:** Data curation (supporting); Formal analysis (supporting); Writing – review & editing (equal). **Ina Bergheim:** Conceptualization (lead); Data curation (equal); Formal analysis (equal); Funding acquisition (lead); Supervision (lead); Visualization (equal); Writing – original draft (equal); Writing – review & editing (equal).

## Supporting information

Figure S1‐S5

Table S1‐S3

## Data Availability

The data that support the findings of this study are available from the corresponding author upon reasonable request.
