# Supplementary material for: Alterations of nitric oxide homeostasis as trigger of intestinal barrier dysfunction in non‐alcoholic fatty liver disease
Source: J Cell Mol Med. 2022 Jan 14;26(4):1206–18. doi: 10.1111/jcmm.17175 (PMC8831936; doi:10.1111/jcmm.17175)
Supplement: Supplementary file 1 — Figure S1‐S5 [file JCMM-26-1206-s001.pdf]

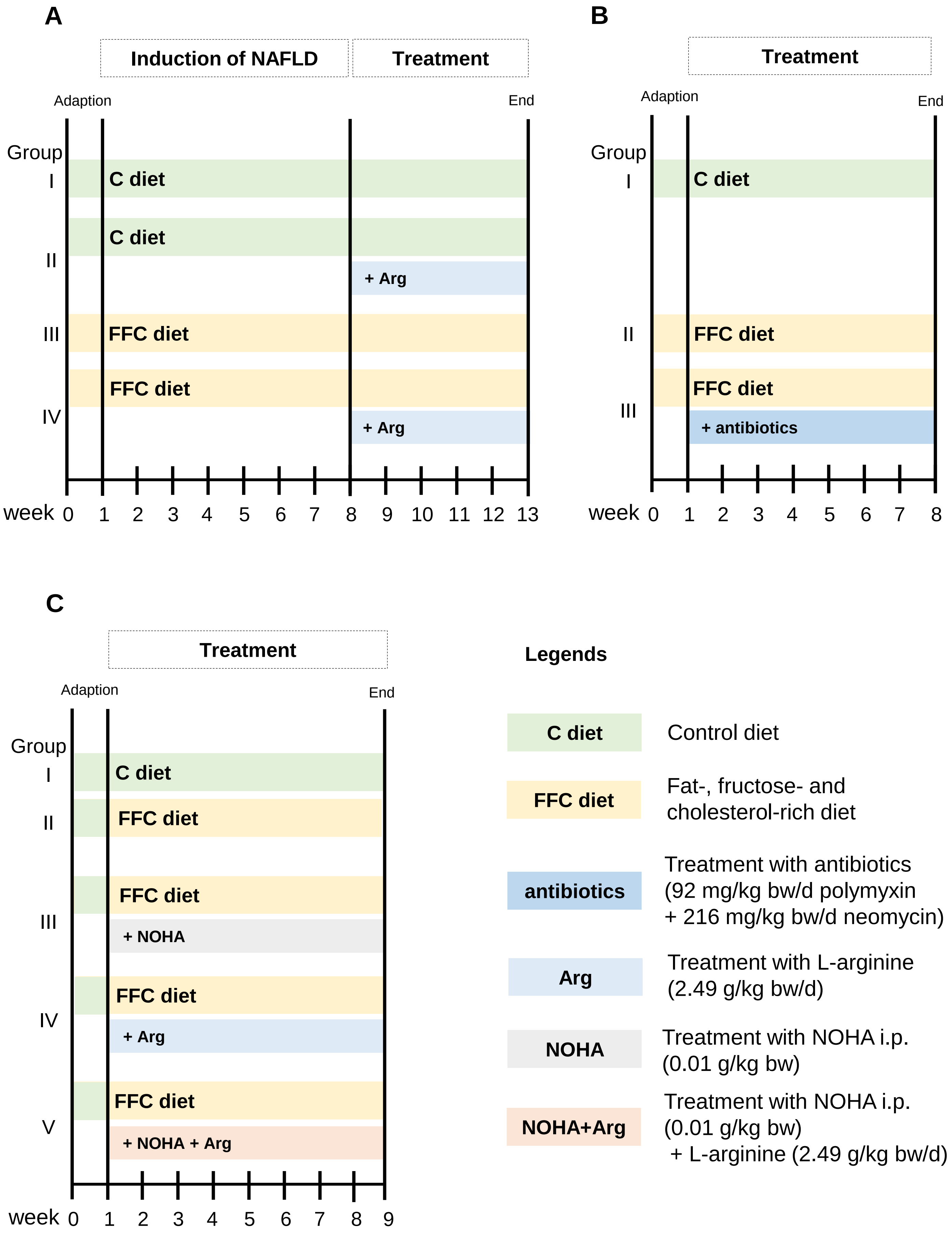

Fig. S1. Study design of (A) intervention trial 1, (B) intervention trial 3 and (C) intervention trial 4. Arg, L-arginine; C, control diet; FFC, fat-, fructose- and cholesterol-rich diet; NOHA, N^{ω}-hydroxy-nor-L-arginine.

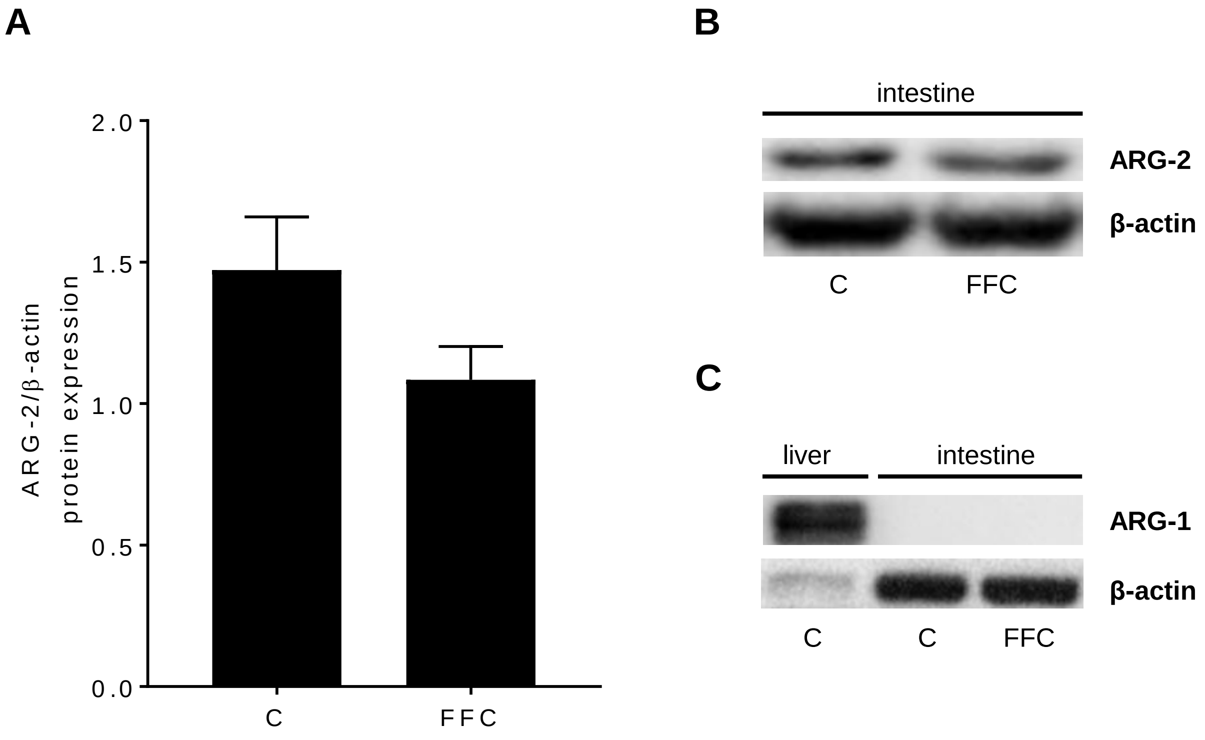

Fig. S2. (A) Densitometric analysis of ARG-2 protein expression in small intestinal tissue and representative picture of (B) ARG-2 protein expression in small intestinal tissue and (C) ARG-1 protein in liver and small intestine of mice fed a C- and FFC diet for 8 weeks. Data are shown as means ± SEM, n=6. ARG-1, arginase 1, ARG-2, arginase 2; C, control diet; FFC, fat-, fructose- and cholesterol-rich diet.

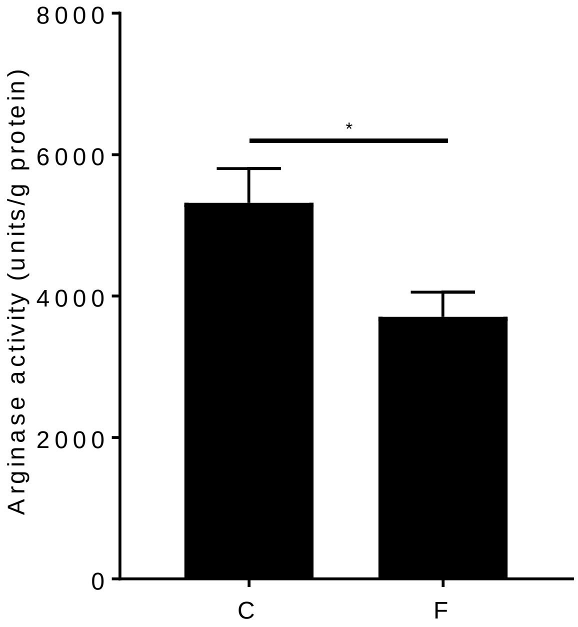

Fig. S3. Arginase activity in small intestinal tissue of mice fed a fructose-enriched drinking water (30 % fructose solution) or plain water for 16 weeks. Data are shown as means ± SEM, n=5-6. C, control diet; F, water enriched with 30 % fructose.

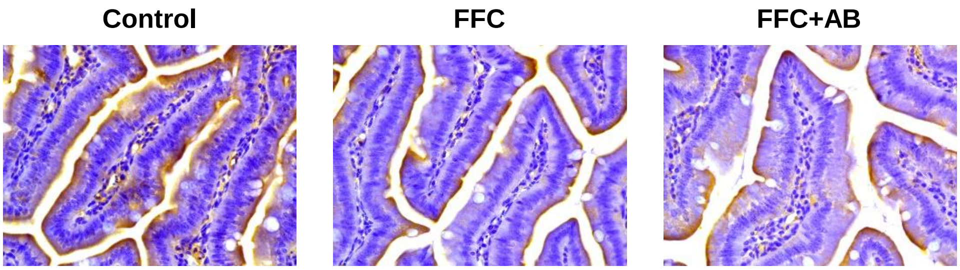

Fig. S4. Representative photomicrographs of ZO-1 staining in small intestinal tissue of FFC-fed mice supplemented with antibiotics for 8 weeks. Magnification 400 x. AB, antibiotics; C, control diet; FFC, fat-, fructose- and cholesterol-rich diet; ZO-1, zonula occludens 1.

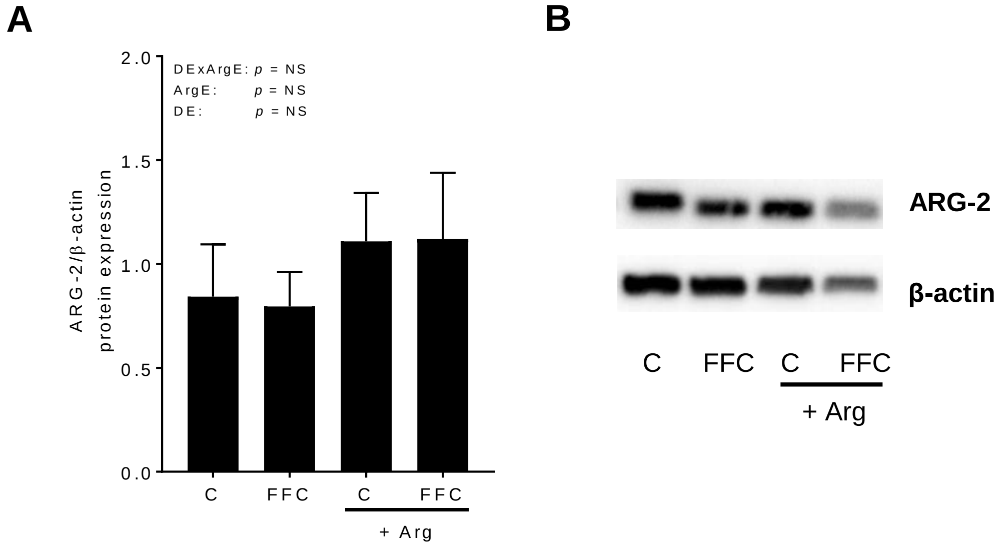

Fig. S5. (A) Densitometric analysis of ARG-2 protein concentration and (B) representative picture of ARG-2 relative protein expression in small intestinal tissue of FFC-fed mice. Data are shown as means ± SEM, n=4-8. Arg, L-arginine; ARG-2, arginase 2; ArgE, L-arginine effect; C, control diet; DE, diet effect; DExArgE, interaction between diet and L-arginine effect; FFC, fat-, fructose- and cholesterol-rich diet.
