# Supplementary material for: Alterations of nitric oxide homeostasis as trigger of intestinal barrier dysfunction in non‐alcoholic fatty liver disease
Source: J Cell Mol Med. 2022 Jan 14;26(4):1206–18. doi: 10.1111/jcmm.17175 (PMC8831936; doi:10.1111/jcmm.17175)
Supplement: Supplementary file 2 — Table S1‐S3 [file JCMM-26-1206-s002.pdf]

Table S1. Characteristics of NAFL patients and age-matched controls.

	C	NAFL
**N**	14	13
**Sex (m/f)**	8/6	6/7
**Age (years)**	44.0 ± 3.1	43.5 ± 4.0
**BMI (kg/m^2)**	22.0 ± 0.6	36.4 ± 3.0 *
**NAS score**		
**(stage 1/2) (N)**	-	7/6
**SAF score**		
**Steatosis (grade 1), (N)**	-	13
**Activity (grade 0/1), (N)**	-	7/6
**Fibrosis (grade 0/1), (N)**	-	7/6
**ALT (U/L)**	17.5 ± 0.9	32.0 ± 3.6 *
**AST (U/L)**	21.7 ± 1.1	27.5 ± 2.3 *
**γ-GT (U/L)**	17.0 ± 1.4	55.1 ± 19.6 *
**Triglycerides (mg/dl)**	71.4 ± 6.0	182.5 ± 24.3 *
**Total cholesterol (mg/dl)**	201.9 ± 8.5	203.7 ± 13.3
**LDL (mg/dl)**	112.9 ± 7.8	122.6 ± 12.5
**HDL (mg/dl)**	74.7 ± 3.0	44.6 ± 2.1 *
**Fasting glucose (mg/dl)**	86.1 ± 1.9	98.8 ± 4.1 *
**Insulin (mU/L)**	5.5 ± 0.5	12.9 ± 2.9 *
**HOMA index**	1.17 ± 0.1	3.3 ± 0.9 *

Data are shown as means ± SEM, n=12-14, $^{*}p<0.05$. γ-GT, gamma glutamyl transferase; ALT, alanine aminotransferase; AST, aspartate aminotransferase; BMI, body mass index; C, healthy controls; HDL, high density lipoprotein; HOMA, Homeostasis Model Assessment; LDL, low density lipoprotein; NAFL, patients with steatosis; NAFLD, non-alcoholic fatty liver disease; NAS, NAFLD activity score; SAF, score for steatosis, activity and fibrosis.

Table S2. Primer sequences used for real-time PCR.

	Forward (5´ - 3´)	Reverse (5´ - 3´)
18S	gtaacccgttgaaccccatt	ccatccaatcggtagtagcg
F4/80	tggctgcctccctgactttc	caagatccctgccctgcact
Il6	ccacgccttccctacttca	tgcaagtgcatcatcgttgttc
Myd88	caaaagtggggtgcctttgc	aaatccacagtgcccccaga
Tlr4	agccattgctgccaacatca	gctgcctcagcagggacttc
Tnfα	cagccaaccaggcaggttct	cctgccacaagcaggaatga

Il, interleukin; Myd88, myeloid differentiation primary response 88; Tlr4, toll-like receptor 4; Tnfα, tumor necrosis factor alpha. Expressions were normalized to 18S mRNA expression.

Table S3. Effect of antibiotic treatment on caloric intake, body- and liver weight and parameter of liver damage in C57BL/6J mice fed FFC.

	Diet groups		
	**C**	**FFC**	**FFC+AB**
**Caloric intake (kcal/g bw)**	0.49 ± 0.0	0.49 ± 0.0	0.47 ± 0.0
**Body weight (g)**	20.3 ± 0.4	21.8 ± 0.2	21.5 ± 0.5
**Liver weight (g)**	0.94 ± 0.1	1.35 ± 0.1	1.28 ± 0.2
**Liver to body weight ratio (%)**	4.6 ± 0.1	6.2 ± 0.1	5.9 ± 0.2
**AST (U/l)**	49.8 ± 4.6	147 ± 43.2	71.3 ± 12.8 *

Data are shown as means ± SEM, n=7-8, *p<0.05 compared with FFC-fed mice. AB, antibiotics; AST, aspartate aminotransferase; C, control diet; FFC, fat-, fructose- and cholesterol-rich diet.
